# A machine learning framework for accurately recognizing circular RNAs for clinical decision-supporting

**DOI:** 10.1186/s12911-020-1117-0

**Published:** 2020-07-09

**Authors:** Yidan Wang, Xuanping Zhang, Tao Wang, Jinchun Xing, Zhun Wu, Wei Li, Jiayin Wang

**Affiliations:** 1 School of Computer Science and Technology, Shaanxi Engineering Research Center of Medical and Health Big Data, Xi’an Jiaotong University, Xi’an, China; 2 The Key Laboratory of Urinary Tract Tumors and Calculi, Department of Urology Surgery, The First Affiliated Hospital, School of Medicine, Xiamen University, Xiamen, China

**Keywords:** RNA-seq data analysis, Circular RNA, Detection method, Machine learning, High precision

## Abstract

**Background:**

Circular RNAs (circRNAs) are those RNA molecules that lack the poly (A) tails, which present the closed-loop structure. Recent studies emphasized that some circRNAs imply different functions from canonical transcripts, and further associated with complex diseases. Several computational methods have been developed for detecting circRNAs from RNA-seq data. However, the existing methods prefer to high sensitivity strategies, which always introduce many false positives. Thus, in clinical decision-supporting system, a comprehensive filtering approach is needed for accurately recognizing real circRNAs for decision models.

**Methods:**

In this paper, we first reviewed the detection strategies of the existing methods. According to the features from RNA-seq data, we showed that any single feature (data signal) selected by the existing strategies cannot accurately distinguish a circRNA. However, we found that some combinations of those features (data signals) could be used as signatures for recognizing circRNAs. To avoid the high computational complexity of the combinational optimization problem, we present CIRCPlus2, which adopts a machine learning framework to recognize real circRNAs according to multiple data signals captured from RNA-seq data. By comparing multiple machine learning frameworks, CIRCPlus2 adopts a Gradient Boosting Decision Tree (GBDT) framework.

**Results:**

Given a set of candidate circRNAs, reported by any existing detection tool(s), the features of each candidate are extracted from the aligned reads. The GBDT framework can be trained by a training dataset. By applying the selected features on the framework, the predictions on true/false positives are reported. To verify the performance of the proposed approach, we conducted several groups of experiments on both real RNA-seq datasets and a series of simulation datasets with different preset configurations. The results demonstrated that CIRCPlus2 clearly improved the specificities, while it also maintained high levels of sensitivities.

**Conclusions:**

Filtering false positives is quite important in RNA-seq data analysis pipeline. Machine learning framework is suitable for solving this filtering problem. CIRCPlus2 is an efficient approach to identify the false positive circRNAs from the real ones.

## Background

Circular RNA, a new star in RNA family, is different from the canonical transcripts, in which they are characterized by a back-splicing event where the downstream 3′ splicing ‘tail’ joins back with the upstream 5′ splicing ‘head’ to form a circular RNA structure [1–3]. Accumulated studies have reported and proved that circRNAs regulate multiple genetic information flows and undertake various biological functions [4, 5]. For instance, circRNA is observed a negative correlation of global abundance and proliferation in some colorectal cancer cases [6]. Nonetheless, the functions of the majority of circRNAs still remain unknown and thus raise the researchers’ curiosity to further investigate them. To solve the circRNA mystery, identifying circRNAs from various samples becomes a basic step for any further analyses [7]. Along with the increasing popularity of RNA sequencing (RNA-seq), using the computational approaches to detect circRNAs from enormous RNA-seq reads become the major strategy [2, 8]. In the past several years, a series of detection algorithms have been developed. Based on our best knowledge, the existing algorithms mainly rely on the back-spliced junction reads (BSJ reads), which are considered as a key feature (data signal) in circRNA detection. However, the occurrence of BSJ signal is not sufficient to prove the circularity of the transcript of origin; non-BSJ reads may also yield similar signals because of either sequencing/mapping errors or the existence of mutations or repetitive sequences. The existing detection strategies often encounter the difficulties on differentiating BSJ reads from the non-BSJ ones, and may lead to high false positive rates.

Although the existing detection algorithms are distinct with each other in detecting and/or filtering strategies, they always share some basic ideas, which are summarized here first. In general, these algorithms often use one or more features here: 1) PEM signal: The candidate junction read is considered to indicate a circRNA only when the mapping position of its paired is within the putative circRNA region, according to the reference genome [9]; 2) Back-splicing signal: Two segments of one read are mapped to the reference genome respectively. In addition, if the mapping positions on the reference genome present a reversed upstream (or downstream) order, then this read is called a back-spliced junction (BSJ) read. The existing algorithms adopt different strategies to search BSJ reads. For example, CIRI [9] mainly utilizes the paired chiastic clipping (PCC) signals, which are captured from the aligned reads find_circ considers a junction read to be a BSJ read only if its front and back parts can be aligned on the reference genome in a reversed order. Our previous method, CIRCPlus [10], identifies a set of BSJ reads spanning the same breakpoint by comparing the local similar sequence of each pair of them. 3) GT-AG signal: The GT-AG signals are the major structural signals in eukaryotic transcriptions, and often used for de novo circRNA detections [9].

A couple of comparison studies on circRNA detection algorithms are conducted on both artificial and real RNA-seq datasets, which also discuss the multiple aspects influencing the performance [11, 12]. Each algorithm presents both strength and weakness, and no one outperforms others on obtaining both a higher sensitivity and specificity [11–13]. For example, CIRCPlus had higher sensitivity than other algorithms because it introduces a strategy to merge the locally re-aligned fragments, which is beneficial for re-using the biased spanning BSJ reads. However, such a comprehensive detection strategy may lead to relatively high false discovery rates (FDR) [10]. The difficulties in accurately identifying circRNA may largely fall into one of these computational challenges: 1) the expression levels of circRNAs are usually lower than the linear RNAs that share the same exons or fragments, and thus the reads from linear transcripts interfere the detection algorithms, as the data signals of circular junction are diluted [14, 15]. If minor reads support a circular junction, it is difficult to identify the real circRNAs from the putative ones only by limited mapping quality and supporting reads [9, 11]. 2) Although most of the algorithms utilize PEM signal to reduce false positives caused by ambiguous mapping, we may still capture false positive BSJ reads that originate from the linear RNAs when both the orientation and library insert length, by chance, coincide with the genomic region of a putative circRNA. 3) In addition, when multiple filters are adopted by the algorithms to achieve a lower FDR, each filter is usually preset a stringent threshold. It is hard to obtain a best practice for setting those thresholds. The filters may exist in name only when the thresholds take into account the first case, while it may introduce false negatives when they consider the second one.

In this paper, we developed a new approach, called CIRCPlus2, to recognize circRNAs from a set of candidate circRNAs and the RNA-seq data. Different from the existing circRNA detection algorithms that use multiple hard filtering strategies, CIRCPlus2 adopts a machine learning framework to obtain a more comprehensive combination on multiple features and their thresholds. Moreover, there are still some ambiguous cases according to the features proposed in the existing algorithm. For example, the algorithm cannot determine a junction with low mapping quality or limited supporting reads is from a circRNA or not. Thus, additional features are also suggested in our method, such as the consistency, integrity and the read depth of which are mapped at the upstream and downstream splice sites of candidate circRNAs. CIRCPlus2 uses a Gradient Boosting Decision Tree (GBDT) framework, which is suggested as more efficient and requires less training data. To the best of our knowledge, it is the first tool using the machine learning approach to distinguish the true and falsely detected circRNAs from the candidate set. According to the experiments, CIRCPlus2 can process the balanced performance with high sensitivities and low FDRs for circRNA detection on different sequencing coverages, different read lengths and other important configurations.

## Methods

Here, we propose CIRCPlus2, an algorithm designed to precisely differentiate the circRNAs from a set of candidate circRNAs using an efficient machine learning framework. CIRCPlus2 does not detect circRNAs. Instead, the candidate set can be detected by any of the existing methods. It should be noted that, in order to obtain a better performance, it is suggested to get a more complete candidate circRNA set by extending the outputs of the chosen detection algorithm with less stringent filtering thresholds.

Besides the candidate set, CIRCPlus2 requires two input files, a FASTA formatted reference sequences and the Sequence Alignment/Map (SAM) alignment file corresponding to the candidate set. The BAM file is suggest to be generated by BWA-MEM, which implements a local alignment and outputs both the primary and other major alignments for all segments of a query read that are separately mapped to the reference genome.

**Fig. 1:**
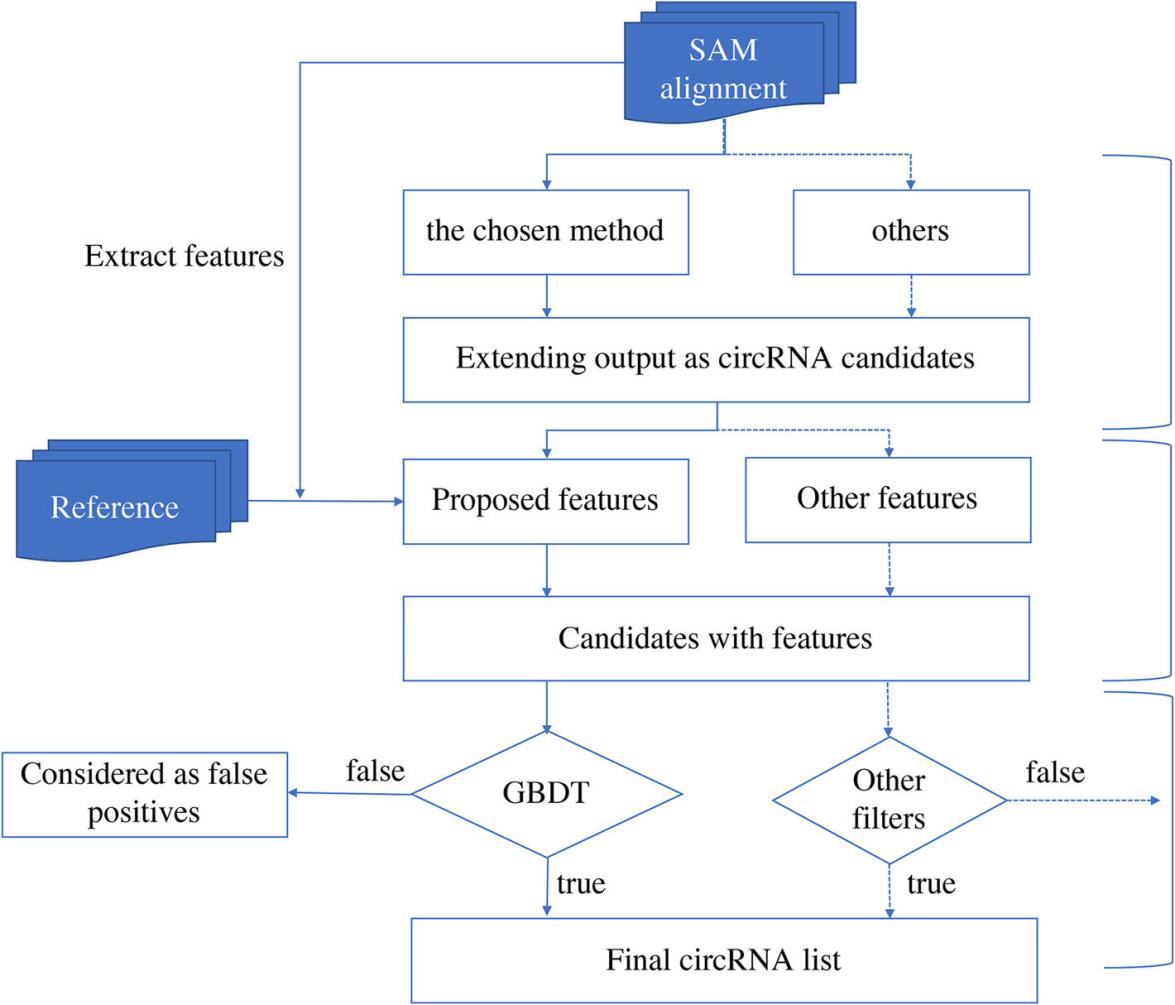
The CIRCPlus2 pipeline for recognize circRNAs from RNA-seq data

Overall, CIRCPlus2 consists of three major steps: 1) The candidate circRNAs are collected from the outputs of one or multiple existing algorithms; 2) The features are extracted for each candidate circRNA and put into a Gradient Boosting Decision Tree (GBDT) framework, which has been already trained by a labelled training set; 3) The GBDT framework reports the label of each candidate circRNA, where it is classified into either a real circRNA or a false positive one. CIRCPlus2 can be extended by allowing the import the raw circRNAs which are detected by other/additional features or by incorporating other filters. The overall workflow of CIRCPlus2 is shown in Fig. 1; each step will be described in detail respectively.

### Extract features of CircRNAs

**Fig. 2:**
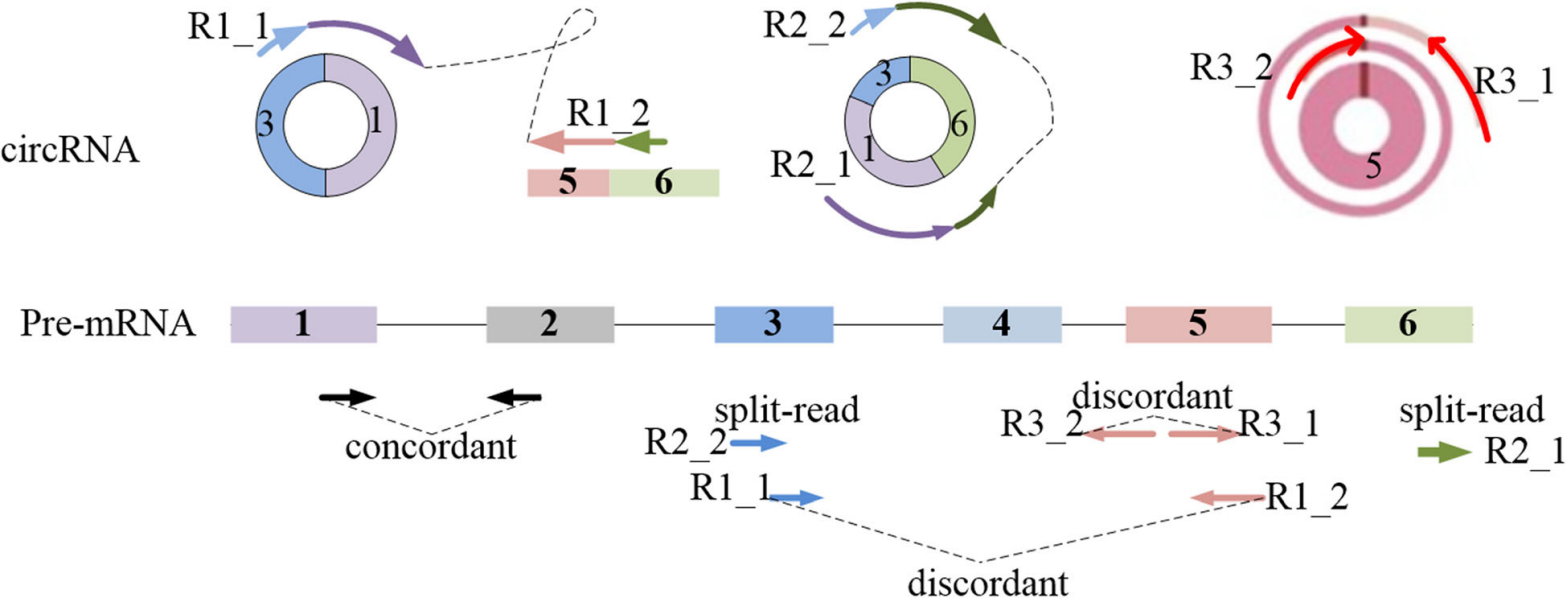
The examples of different types of circRNAs and the related features. Split-read: a read that span one or multiple covalent linkages. Discordant read pair: a read pair with both ends mapped, but the locations are too far from or close by each other comparing to the library insert size. FSJ: forward-spliced junction read. BSJ: back-spliced junction read

Collecting features is the key step for filtering out false positive circRNAs. We intend to extract all the features which can effectively and comprehensively represent the properties of circRNAs. We not only consider the features that indicate the presence of circRNA, but collect the features that imply the absence of circRNAs as well. First, when a circular structure occurs across a genomic region, various features of the circRNA can be captured from the reads. Besides the features introduced in the Background section, we find additional features which are also affected, including the numbers of read-pairs, split-reads and read-depth, etc. Here, we present some interesting observations. As shown in Fig. 2, a circRNA has two breakpoints (i.e. the left and right genomic boundaries). For a given circRNA, it can be represented as [*brk*_1_, *brk*_2_], where *brk*_1_, *brk*_2_ are the left and right breakpoints of the circRNA. It should be further extended by a detection range added to left and right breakpoint, respectively. Here, we select *range* = *μ* + 3*σ*, where *μ* and *σ* are the mean and standard deviation of the library, respectively. The paired-end reads collected in the areas of [*brk*_1_ − *range*, *brk*_1_ + *range*] and [*brk*_2_ − *range*, *brk*_2_ + *range*] are often influenced by the existence of circRNAs. Other types of circRNAs are also shown in Fig. 2 and five types of related features are able to be calculated from the mapped reads.

We further refine these features and give out a series of more detailed features. Table 1 lists all the symbols used by feature extraction of this work, and they are explained in subsequent sub-sections.

**Table 1:** List of features

Category	Symbol	Meaning
Insert size of read pair	Concord	Concordant pair
	Discord	Discordant pair
BSJ reads	Mapping_quality	Mapping quality
	Support	Supporting read count
Breakpoint	l	Left breakpoint
	r	Right breakpoint
Mapping situation in breakpoint	SM	CIGAR value in the form of xS/HyM
	MS	CIGAR value in the form of xMyS/H
	SMS	CIGAR value in the form of xS/HyMzS/H
Splicing signal	GTAG	GT-AG signal
Depth	Depth	Average read depth
	Cov	Average read base count
Region (circRNA length)	Up	Up of circRNA region
	Down	Down of circRNA region

#### Refined features with concordant and discordant pair-end read

The discordant and concordant paired-end reads signals have been generally used in many variation detection methods. Discordant paired-end reads also support the existence of circRNA. For a given candidate, we collect the read pairs which encompass the potential circRNA region. The insert sizes of the read pairs may be discordant with the library insert size. We define a threshold value of *m + 3v*. Here, *m* and *v* are the mean and standard deviation of library insert size respectively. For any RNA-seq data with well quality-controlled library, the insert sizes often follow a normal distribution. A mapped read pair is considered to be discordant if its mapped insert size is larger than this threshold. Otherwise, we consider that this read pair is concordant. Figure 2 shows discordant read pairs occurred on both circRNA breakpoints.

CircRNAs often drive the local distributions different from the normal one. For example, 1) if there is a read from the region of circRNA and its mate read comes from the flanking region of the circRNA, it increases the insert size when mapped these reads to the reference genome. In another case, 2) if the length of a circRNA is smaller than the insert size or even the read length, it could narrow the insert size. According to the data analysis, we also observe that the circRNA may also drive the local distribution of insert sizes different from the linear RNA drives. Thus, we count the numbers of the concordant and discordant read pairs encompassing the circRNA breakpoint, which are marked as concord and discord. In detail, the discordant or concordant paired-end reads should be collected in the areas of [*brk*_1_ − *range*, *brk*_1_ + *range*] and [*brk*_2_ − *range*, *brk*_2_ + *range*].

Another important features are related to the coverage. Coverages of training and testing datasets may be quite different. Coverages around different circRNAs may also be significantly different. Suppose that there is one circRNA, whose local coverage is quite low. Even though there are only few of discord, these read pairs still strongly support the presence of the circRNA. On the contrary, if the coverage around another circRNA is quite high, a small number of discord should not be a strong signal of the presence of a circRNA. Thus, another four features are collected, and named as discord_brk1, discord_brk2, concord_brk1, concord_brk2, respectively.

#### Refined features according to read depth and bed coverage

Read depth is a widely used feature for sequencing data, which refers to the number of reads mapped to a particular site or genomic region. A large proportion of circRNAs have relatively low abundance compared with their linear counterparts [14, 15]. Therefore, the read depth may indicate the existence or absence of circRNAs. Moreover, the left and right breakpoints should be treated separately, which could contribute to recognize the false positive circRNAs with inaccurate breakpoint locations. A region on the reference genome with a smaller average mapped read depth or an extremely higher one than the expected read depth may support the existence of circRNAs. We calculate average read depth of one region via

$$ depth={\sum}_{i=1}^{l_d}\left({d}_i\right)/{l}_d $$

Where *l*_*d*_ is the length of the region and *d*_*i*_ is the mapped read depth of any position *i*. SAMtools can compute the *d*_*i*_ values at each position across the region. In addition, we also calculate the total read base count (i.e. the sum of per base read depths) for each genomic region specified in the supplied BED file by SAMtools. The bed coverage of one region can be calculated by

$$ {\mathit{\operatorname{cov}}=\sum}_{i=1}^{l_d}\left({c}_i\right)/{l}_d $$

Where *l*_*d*_ is the length of the region and *c*_*i*_ is the mapped read base count of any position *i*. The average read depths and bed coverages for a circRNA region and the upstream and downstream regions can be collected for eight features, which are cov_brk1_Up, cov_brk1_Down, cov_brk2_Up, cov_brk2_Down, depth_brk1_Up, depth_brk1_Down, depth_brk2_Up, depth_brk2_Down, respectively.

#### Refined features of partially mapped reads and one-end splitting reads

Split-read is another important feature for detecting and recognizing circRNAs. Split-reads are the reads spanning the splice site and may be clipped mapped to the reference genome. When it is properly mapped, each read has three possible cases, which are fully mapped, clipped mapped or unmapped. Fully mapped means a read is mapped as a whole, which supports the absence of any circRNAs at the mapping locations. Clipped mapped means a read cannot be mapped as a whole, but one segment (prefix or suffix) is able to be mapped (as soft or hard clip), which supports a potential presence of a circRNA. Clipped mapped reads are usually helpful to find the exact breakpoints of the circRNAs. In addition, unmapped reads means there are no suitable mapping locations on the reference genome, regardless of how the reads are splitted, which can be temporary ignored in circRNA detection. Therefore, we focus on extraction the feature of split-read in this step.

Split-read of different circRNAs often have different alignment features, and present in different CIGAR values. A typical junction is separately mapped to the reference in a corresponding two-segment style, as shown in Fig. 2. The split reads locate at 5′ splice site show the CIGAR values in the form of xS|HyM, while the split reads locate at 3′ splice site show the CIGAR values in the form of xMyS|H. In addition, some circRNAs have complex alignment features. These junction reads may be mapped in an inconsecutive order to the reference genome. For example, in a three-segment style, the CIGAR values present the alignment features in the form of xS|HyMzS|H at 5′ splice site and 3′ splice site. Here, CIRCPlus2 first extracts the split reads from the BAM file, and then calculates the read counts with different CIGAR types each of which implies a corresponding alignment pattern. Therefore, six features around the left and right breakpoints of a candidate circRNA are obtained, which are represented as SM_l, MS_l, SMS_l, SM_r, MS_r, SMS_r, respectively.

#### Refined features of GT-AG signal

GT-AG signal is the major splicing signal in eukaryotic transcription and is used for circRNA detection in many existing algorithms. CIRCPlus2 loads the reference sequences to check whether the AG and GT dinucleotides (or reverse complementary disnucleotiides CT and AC) have the flank segments of a junction (Fig. 2). Due to the ambiguity of the junction boundaries identified from the alignments, GT and AG signals are accepted if both of the deviations are acceptable from the tentative boundaries along the reference sequence. The candidate junction reads not supported by splicing signals or exon boundaries are still further detected even they are lack of the GT-AG signals.

#### Refined features of BSJ read

Finally, other features including the supporting read count and average mapping quality also contribute to distinguish the candidates. We analyze the basic read mapping situations in, up and down (5' or 3' direction) of the circRNA regions. The supporting read count means the number of back-spliced junction reads which are detected by a circRNA detection algorithm. Meanwhile, the mapping quality also has contribution to call the real circRNAs. The higher the mapping quality presents, the more accurate the alignment achieves. Especially for some junction reads, which have a much shorter segment flanking the junction compared to the other segment, the short segment (< 19 bp using the default parameter of BWA-MEM) is always ignored by the aligner to prevent the algorithm from further reporting multiple mapping locations or erroneous mapping locations. Such junction reads may have quite low mapping quality in the SAM alignment. Therefore, the BSJ reads have higher mapping qualities are more reliable for recognizing circRNAs. Here, we calculate the average mapping quality of one breakpoint by

$$ Mapping\_ Quality={\sum}_{\mathrm{i}=1}^{l_d}\left({m}_i\right)/{l}_d $$

Where *m*_*i*_ is the mapping quality of the supporting BSJ read and *l*_*d*_ is the supporting read count. Existing circRNA detection algorithms usually set a high and overall cutoff for the supporting read count and mapping quality of BSJ reads to filter false positives, while in CIRCplus2 we divide them into four features, which are Support_l, Mapping_Quality_l, Support_r, Mapping_Quality_r, for the left and right breakpoints respectively.

### Gradient boosting decision tree framework

After collect the proper features that can distinguish these two types, the next step is the machine learning framework chosen for the classification problem. Gradient boosting is a widely used machine learning framework for classification problems, which produces a prediction model in the form of an ensemble of a series of weak prediction models, typically the decision tree models. It builds the model in a stage-wise fashion like other boosting methods do, and then it generalizes them by allowing optimization of an arbitrary differentiable loss function. Notably, Gradient Boosting Decision Tree (GBDT) is the most popular algorithm in gradient boosting model family, which is used for classifying or regressing the data by implementing an additive model and continuously reducing the residuals generated in multiple iterations. Through multiple iterations in GBDT, each weak classifier is trained on the basis of the residuals of the previous classifier to improve the accuracy of the final classifier. GBDT is an ensemble learning algorithm that does not require the data preprocessing and is reported to be less sensitive to the outliers. For linear inseparable data, GBDT often achieves good performance than other popular machine learning models, especially in prediction accuracy. In addition, as a tree-based framework, GBDT is suggested to be generally more resistant to the mass noise, which is suitable for the candidate circRNA datasets. Therefore, GBDT is a good choice for the classification problem of candidate circRNAs.

Given a candidate circRNA, all of the features are collected and the values are extracted. Consider a vector, where each element represents a feature. Then, for each candidate circRNA, it should be represented as a point in a *k* dimension space, where *k* is the number of features and each feature indicates a dimension. An optimal panel is learned from the training data, and the framework is then used to classify the testing data.

As a supervised machine learning framework, a training set is needed to train the GBDT framework. The training set consists of thousands of candidate circRNAs with labels. Each training candidate has a binary label, where the label denotes the candidate is either a real circRNA or a false positive. It is not difficult to find a training set from public database. On the other hand, simulation datasets are more helpful if the existing training sets are different from the testing set. Here, we use CIRI-simulator, a popular software, to generate the simulation datasets. CIRI-simulator requires a reference and an annotation file. We use chromosome 1-22 from hg19 as reference genome and its GTF annotation file (Gencode version 18) is downloaded from [16]. We use default settings of CIRI-simulator to generate numerous circRNAs and linear RNAs with different lengths. Some transcripts share the exons. We applied CIRI2, find_circ and CIRCPlus to detect candidate circRNAs. These candidates are further labeled according to the benchmark data provided by CIRI-simulator.

### Collecting candidates by existing tool

There are several existing tools can be used to detect circRNAs to get the candidate circRNAs. The existing state of the art tools, e.g. CIRI, CIRI2, find_circ, CIRCPlus can be used. Certainly, this step is modifiable so that users can also use other tools. Each tool has a set of internal parameters, whose settings usually significantly affect the outputs. In order to obtain a high sensitivity on circRNA detection, we suggest to keep a more comprehensive set of the candidate circRNAs, although it usually introduce more false positives.

In addition, as a partner tool, here we introduce the process that CIRCPlus2 collects candidate circRNAs from the outputs of CIRCPlus, a circRNA detection algorithm previously published. Overall, CIRCPlus2 uses the paired similar sequence (PSS) signals which are captured by CIRCPlus to obtain the candidate circRNAs without other filter steps. It is previously reported that the PSS signal can identify more BSJ reads, some of which are usually ignored or misclassified by the existing algorithms. Moreover, the PSS signal has advantages over other approaches on detecting different types of circRNAs with better performance on different features of data (e.g. low or high coverages, short or long read lengths) [10]. Briefly, a pair of reads that indicate a circRNA junction should be both aligned to the reference genome with a local similar sequence. The CIGAR values reflect the junctionare in the form of upstream × 1S/Hy1M and downstream × 2My2S/H, respectively, where × 1, × 2, y1 and y2 represent the numbers of mapping (M), soft clipping (S) or hard clipping (H) bases, respectively. Some specific types of circRNAs, such as the short-exon flanking circRNA and small circRNA, may have different CIGAR values for one junction read. These junction reads may be inconsecutively mapped to the reference genome in a three-segment style and bring the CIGAR values in another form of upstream or downstream xS/HyMzS/H. Thus, in these cases, there isusually a pair of junction reads located in the upstream and downstream splice site of the circRNA, which often have a local similar sequence named paired similar sequence signal. As the PSS signal is not restricted by the read length or mapping segment counts and is also independent with the annotation, it should be more sensitive and reliable for the junction detection. CIRCPlus2 searches the CIGAR values from the pairs of reads. If it is considered to be located in upstream and downstream splice site of a circRNA as described above, CIRCPlus2 then checks the strand information and mapping locations. If the pair of reads are aligned to the same chromosome, and both the strand and insert-size are reasonable, the pair of reads are considered as the candidate junction reads with positive PSS signals. Because the pair of reads represent the boundaries where all of the reads from the same circRNA are mapped, a candidate junction read is considered to indicate a circRNA only when its paired read is mapped within the region of the putative circRNA range on the reference genome. If both of the conditions are satisfied, the region is considered as a  candidate circRNA.

### Classification with collected features

For the machine learning framework, a 23-dimension vector is extracted for each candidate circRNA, where each dimension represents a feature. In particular, we use GBDT to train the models, and then use the trained model to filter false positive circRNAs. The training dataset need to be labeled and scaled, and then a grid search and a 10-fold cross validation are suggested to find the optimal parameters The Random trees kernel function is used. CircRNAs classified as true ones are the output of CIRCPlus2.

In summary, we proposed the CIRCPlus2 to recognize real circRNAs according to multiple data signals captured from RNA-seq data combined with CIRCplus, these two algorithms are able to balance the performance with high sensitivity and low FDR. In CIRCPlus, certain BSJ reads are detected based on PSS signals due to the split alignment strategy of BWA-MEM, the short splice reads may introduce false positive BSJ reads. Thus, CIRCPlus compares the supporting read counts and the mapping qualities of the reads to filter the false positives. However, these threshold-based filtering strategies are difficult to preset and optimize, and thus often loss sensitivities because of the structure complexity of circRNAs, expression levels, mutations, which may expose different features spanning the junctions. In contrast, CIRCPlus2 achieves this by a machine learning framework, which is able to handle more complicated cases.

## Results and discussion

To validate the performance of the proposed approach, we first compared CIRCPlus2 to CIRI2, CIRI, find_circ and CIRCPlus on a set of artificial datasets with different configurations. We also compared the outputs of CIRCPlus2 with the outputs that were identified on the resistance samples with RNase R treatment which was known to specifically enrich circRNAs. The raw sequencing data was mapped by BWA-MEM under default parameters. In the following experiments, two metrics, sensitivity and precision, were calculated for evaluations. The sensitivity and precision are defined as:

$$ Sensitivity=\frac{TP}{TP+ FN} $$

$$ Precision=\frac{TP}{TP+ FP} $$

Where TP denotes the number of true positives, FP denotes the number of false positives and FN denotes the number of false negatives. To evaluate the performance on balancing the sensitivity and precision, F1-score is also employed, which is calculated by the following formula:

$$ F1- score=\frac{2\times Sensitivity\times Precision}{Sensitivity+ Precision} $$

### Generating simulation datasets

CIRI-simulator is a specific simulation tool for non-canonical transcripts. Here, we used CIRI-simulator to generate the simulated reads and evaluate the performance of CIRCPlus and CIRI2. CIRI-simulator requires two input files: a FASTA formatted reference sequence and a GTF or GFF formatted annotation file. A list of simulated circRNAs and the FASTQ formatted files are then generated. The list is the benchmark for performance evaluation, while FASTQ formatted files are the inputs of the detecting algorithms. The parameters, including the read length, read depth (for circRNAs and linear RNAs, respectively), sequencing error rate, and insert size, can be customized by users.

To test the performance of CIRCPlus2, we first generated the simulation datasets under different configurations. We selected the read lengths of 60, 100, 125 and 150 bp, and altered the average read depths of 10-, 30- and 50-fold to simulate sequencing reads, respectively. For each dataset, the read amount was determined by the sequencing coverage and read length. As it is reported that fusion events in circRNAs are rare, we applied the whole hg19 genome as the reference sequence, but simply used chromosome 1 (length of 249,250,621) to generate simulated sequencing data. In detail, chromosome 1 from hg19 and its GTF annotation file (Gencode version 18) were downloaded from [16]. For each dataset, the outputs were compared to the list to calculate the sensitivity and precision using a custom script. It has been demonstrated that CIRCPlus has almost the highest sensitivities over the other de novo methods [10]; therefore, we concentrate on the improvement on the precisions and comprehensive performance in the following experiments. We evaluated the performance from the following aspects.

### Analysis of comprehensive performance of classification model

#### Detection performance under different read coverages of linear transcripts

**Fig. 3:**
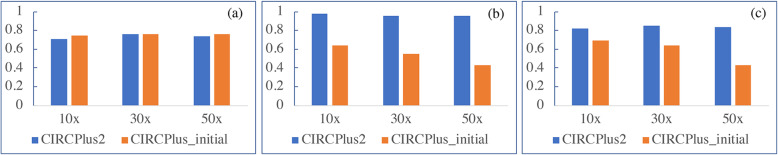
**a** Sensitivity analyses under different linear transcripts coverages (the read length was fixed to 100 bp). **b** Precision analyses under different linear transcritps coverages. **c** F1-Score analyses under different linear transcripts coverages

We first focused on how the classification model improved the detection performance under different read depths of linear transcripts. Paired-end reads (read length of 100 bp) were generated from the reference genome with an increasing average read coverages of linear transcripts, which altered from 10-, 30- to 50-fold, while the average read coverage of circRNA transcripts was kept on 10-fold. The sensitivity, precision and F1-Score of CIRCplus2 and CIRCplus_initial (the initial detection results for CIRCplus2 without the classification step) were calculated for each dataset. The sensitivity of CIRCplus and CIRCplus_initial were shown in Fig. 3a, they were remained stably around 75% when the average sequence coverages of linear transcripts varied ranging from 10- to 50-fold. Notably, there was almost no loss of sensitivities of CIRCplus2 compared to CIRCplus_initial (less than 3% in most of the cases), which suggested that the trained model could accurately classify the candidate circRNA set of CIRCplus_initial into true positives and false positives. At the same time, the precision of CIRCplus2 and CIRCplus_initial were quite different, as shown in Fig. 3b. CIRCplus2 held the higher precisions under different read coverages of linear transcripts than CIRCplus_initial. It also could be seen that the precisions of CIRCplus_initial were largely affected by the increasing of the read coverages of linear RNA, where the precisions decreased from 64 to 43%. On the other hand, CIRCplus2 improved the precision up to 98% with the help of the classification model. Therefore, the true and false positives in the candidate circRNA set could be accurately distinguished by the trained model of CIRCplus2 and obtained higher precisions without significant loss of sensitivities. From Fig. 3c, CIRCplus2 increased the F1-Score compared to CIRCplus_initial from 60 to 84%.

#### Detection performance under different read lengths

**Fig. 4:**
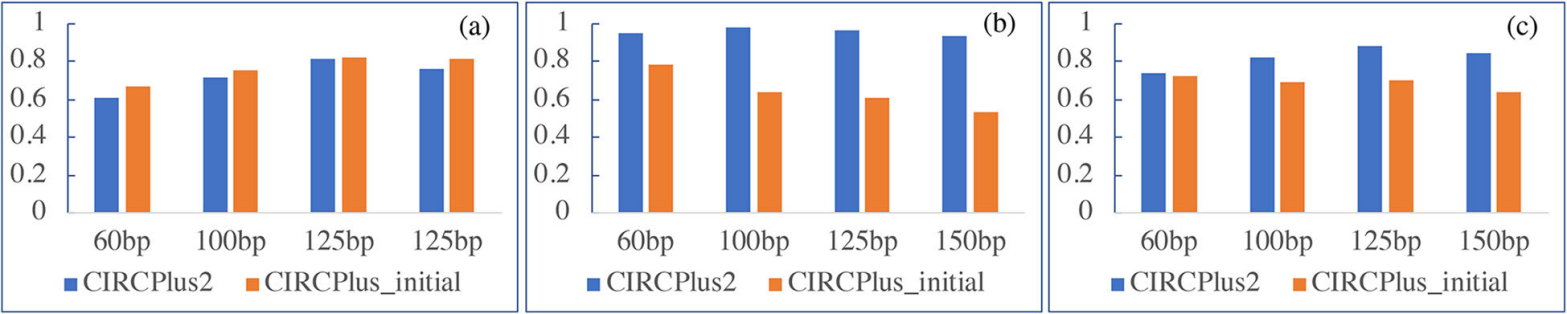
**a** Sensitivity analyses under different read lengths. **b** Precision analyses under different read lengths. **c** F1-Score analyses under different read lengths

The detection improvement of CIRCplus2 on variable read lengths was also analyzed in a group of experiments. We increased the read lengths from 60 to 150 bp, while the average read depths of circRNAs and linear RNAs were set to 10-fold. The results were shown in Fig. 4. From Fig. 4a, it could be seen that the sensitivities of CIRCplus2 were a little lower than the ones of CIRCplus_initial. The sensitivity losses between CIRCplus2 and CIRCplus_initial became smaller with the increasing of read lengths. In detail, CIRCplus2 maintained a high level of sensitivities (around 80%), which demonstrated that the trained model implemented in CIRCplus2 had satisfied performance on predicting the true positives. At the same time, the precision of CIRCplus_initial decreased a lot with the increasing of read lengths, and it only reached 53% when the read depth was set to 150 bp (Fig. 4b). In comparison, CIRCplus2 still remained a high precision (around 95%) for each dataset after it filtered a large false positives in the candidate set, and it improved the precision up to 93% when the read length was set to 150 bp. In addition, from the F1-Score analysis shown in Fig. 4c, we could conclude that CIRCplus2 improved the comprehension performance of CIRCplus_initial under different read lengths, especially for the datasets with longer read length (above 100 bp). It should be noted that the trained model used in CIRCplus2 had good generalization ability and could distinguish the two types of candidates with different data with a high level of accuracy.

### Analysis of comprehensive performance of detection

#### Detection performance under different read coverages of linear transcripts

**Fig. 5:**
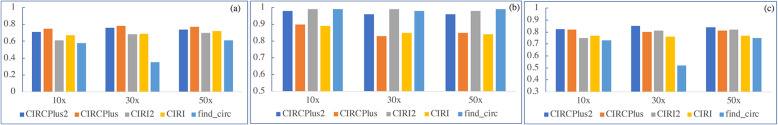
**a** Sensitivity analyses under different read depths of linear transcripts (the read length was fixed to 100 bp). **b** Precision analyses under different read depths of linear transcritps. **c** F1-Score analyses under different read depths of linear transcripts

We then focused on how the expression levels of circRNAs affected the performance of different detecting methods. Paired-end reads were generated from the reference genome with an increasing average read depths of linear transcripts, which altered from 10-, 30- to 50-fold, while the average read depths of circRNA transcripts was kept on 10-fold. For each dataset, we calculated the sensitivity, precision and F1-Score of CIRCPlus2, CIRI2, CIRI, find_circ and CIRCPlus (Fig. 5). For read length of 100 bp, the sensitivity results were shown in Fig. 3a, where CIRCplus and CIRCPlus2 always had a higher sensitivity than others. In detail, the sensitivity of CIRCPlus2 remained stably around 74% when the average read depths of linear transcripts varied ranging from 10- to 50-fold, while CIRI2 was around 66%. The sensitivity of find_circ fluctuated greatly along with the increasing of read depths of linear transcripts, e.g. it only detected 30% circRNAs when the read depths of linear transcripts was 30-fold. Combined with the precision of different detecting methods, shown in Fig. 3b, it was obvious that CIRCPlus maintained the highest sensitivities than others; however, it exposed relatively low precisions. CIRCPlus2 largely improved the precisions of CIRCPlus, which were increased from 80 to 98%, and obtained the similar high precisions as CIRI2. Therefore, it demonstrated that filtering the false positives in the candidate circRNAs by machine learning in CIRCplus2 was more reliable than the strategy of setting a high cutoff value in CIRCPlus. From Fig. 3c, CIRCPlus2 held the highest F1-Score under different read lengths comparing to others. In detail, the F1-Score of CIRCPlus2 remained stably around 84%, at the same time, CIRI2 remained around 79% and find_circ showed a lower performance.

#### Detection performance under different read lengths

**Fig. 6:**
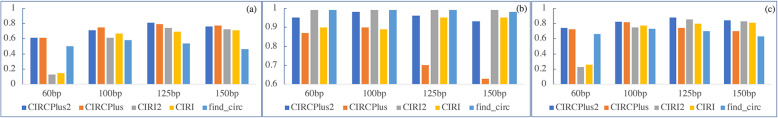
**a** Sensitivity analyses under different read lengths. **b** Precision analyses under different read lengths. **c** F1-Score analyses under different read lengths

We also tested the performance of CIRCPlus2 on variable read lengths and compared to the other four detection methods. In this group of experiments, the read lengths varied from 60 to 150 bp, while the average read depths of circRNAs and linear transcripts were all set to 10-fold. The results were shown in Fig. 6. From Fig. 6a, the sensitivities of different methods were gradually increasing along with the increasing of read length. CIRCPlus2 and CIRCPlus had the higher sensitivities than others and they reported almost the same detection outputs in each dataset. From Fig. 6b, CIRCPlus2 significantly improved the precisions compared to CIRCPlus especially when the read length was 150 bp (63–93%), and the precisions of CIRCPlus2 were almost as high as CIRI2, which still reached above 95%. Thus, CIRCPlus2 greatly improved the precision with no sacrifice on sensitivity compared with CIRCPlus. Specifically, when the read length was set to 60 bp, CIRCPlus2 identified 61% of the pre-set circRNAs, while CIRI2 and CIRI only reached around 13%. CIRCPlus2 was efficient for different read lengths, and still achieved stably high F1-score compared to other methods (Fig. 6c).

According to Fig. 5 and Fig. 6, CIRCPlus2 had the highest F1-Scores in the datasets under different simulation parameter settings across all five algorithms. After applied different simulation configurations, we could say that CIRCPlus2 often had a more comprehensive performance advantage than CIRI2, CIRI, find_circ and CIRCPlus, especially when the reads were trimmed, or the read depths of linear transcripts were higher than the read depths of circular transcripts. Therefore, we could conclude that CIRCPlus2 performed better in balancing the sensitivity and precision under different configurations, and thus it was suggested to have comprehensive performance on circRNA detection.

### Benchmarking CircRNA detection using CIRCPlus2

We used a similar criteria with a previous study [12] to evaluate candidate circRNAs detected with BSJ reads count ≥3 in the datasets without RNase R treatment. In detail, if candidate circRNAs detected by each tool were obviously enriched after RNase R treatment (at least 3-fold increased of BSJ reads count), they were labeled as true positives. In contrast, candidate circRNAs did not detect or largely deplete (with fewer BSJ read counts) after RNase R treatment were labeled as false positives. The ratio of false positives in all of the predictions by each tool was calculated as FDR of the method. We next summed true positives predicted by all tools as the estimated total circRNAs in the datasets. The ratio of true positives detected by each tool in the above total circRNAs was used to evaluate the sensitivity of the method. To compare performances among all methods, we applied a single metric F1 score that simultaneously considered sensitivity and precision of the method.

To quantify the performance improvement of CIRCPlus2 compared with CIRI2, we applied both tools to previously generated RNA-seq data sets of 150 bp HEK293 [17] without RNase R treatment. In detail, the No.1–22 chromosome of the HEK293 dataset was selected for detecting circRNAs (X/Y chromosomes were not considered), and 70% candidate circRNAs (2100 out of 2943) detected by CIRCPlus2 were used for training data, and other 30% candidates were used for evaluating our detecting method. In detail, the detection results of CIRCplus2 were equally divided into three groups, each group was used for training data (900 circRNAs), and the other two groups were used as testing data correspondingly, the classification results of these three testing data were shown in Table 2.

**Table 2:** List of Three Confusion Matrixes

Testing group	Confusion Matrix	
1	297 (TP)	159 (FN)
	97 (FP)	347 (TN)
2	349 (TP)	148 (FN)
	92 (FP)	311 (TN)
3	473 (TP)	155 (FN)
	157 (FP)	358 (TN)

**Fig. 7:**
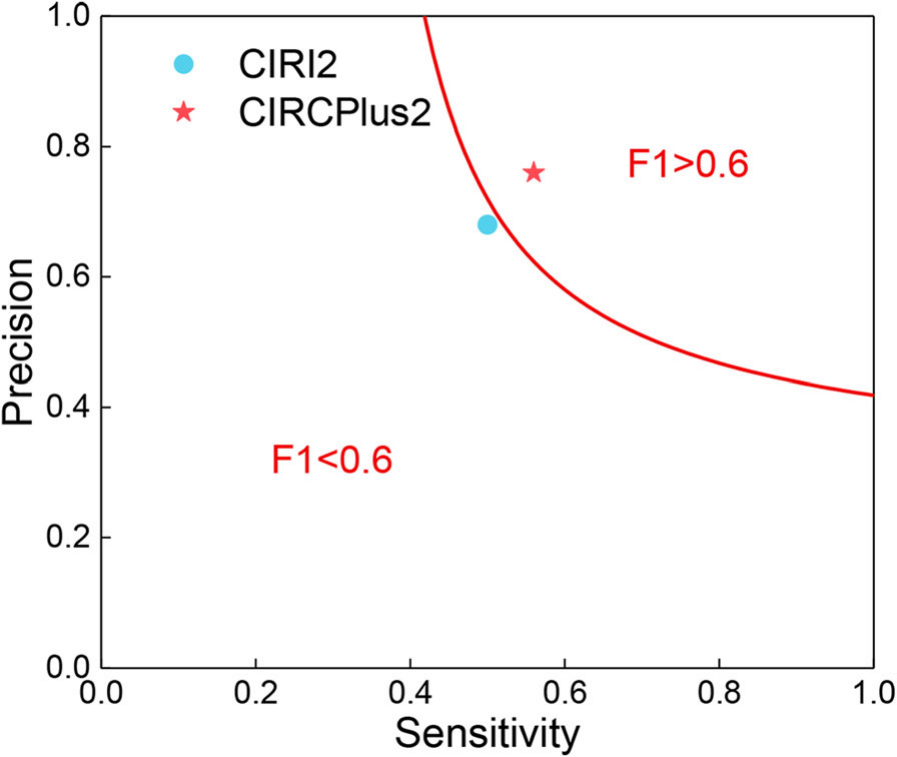
F1-Score of CIRI2 and CIRCPlus2 on the HEK293 dataset

Using the criteria described above, CIRCplus2 totally predicted 1465 circRNAs by combining the results of the three testing sets, and of which 1119 were verified as true positive circRNAs after RNase R resistance evaluation. All true positive circRNAs detected by CIRCplus2 and CIRI2 were used as total circRNA (total 2013 circRNAs). As shown in Fig. 7, CIRCplus2 had a similar sensitivity with CIRI2 and simultaneously achieved a higher precision, thus it could be concluded that CIRCPlus2 had much more balanced performance. To better understand the overall performance of two methods, we defined an evaluation metric, F1 score. As shown in Fig. 7, CIRCPlus2 had the higher F1 score in HEK293 data set than CIRI2. Taken together, the above performance evaluations demonstrated that CIRCPlus2 outperformed CIRI2 on detecting circRNAs.

## Conclusions

In this paper, we proposed a novel algorithm, named CIRCplus2, which focused on the computational problem that identifying circRNAs with a balanced performance of sensitivity and precision from RNA-seq data. It adopts a machine learning framework to accurately identify the true positives in candidate circRNA set which are detected by the existing methods. In detail, CIRCPlus2 first collects the output of one or more existing circRNA detection tools to get a candidate set of circRNAs. Then, CIRCPlus2 captures a series of features for each candidate circRNA from the BAM/SAM file. Here, 23 features are suggested to be related to a circRNA. A GBDT model is trained by the benchmark data. CIRCPlus2 identifies the circRNAs by classifying the candidate circRNA set by the trained model into a subset of true positives and a subset of false positives. In addition, the framework of CIRCplus2 is extensible in each module. CIRCplus2 does not strongly rely on the aforementioned detection tools or the GBDT framework, or is limited to the 23 features. These can be easily extend to additional tools, features and models in applications.

We tested CIRCPlus2 on both simulation datasets and real dataset. A series of experiments show that CIRCPlus2 performed better in most cases comparing to four existing tools. CIRCPlus2 not only shows that the concept of adding a comprehensive filtering step for circRNA detection is effective, but also suggests that the machine learning approach is an effective way of combining features from different sources to distinguish true and falsely detected circRNAs. CIRCPlus2 significantly improves the FDR and F1-Score for identifying the circRNAs under different coverages and read lengths than the existing tools. Therefore, the proposed approach performs both the high sensitivity and high accuracy for the circRNA identifying problem.

## Data Availability

Not applicable.

## Abbreviations

BSJ: Back-Spliced Junction
circRNAs: Circular RNAs
FDR: False Discovery Rate
GBDT: Gradient Boosting Decision Tree
PCC: Paired Chiastic Clipping
RNA-seq: RNA sequencing
SAM: Sequence Alignment/Map
